# Relapse to smoking and health-related quality of life: Secondary analysis of data from a study of smoking relapse prevention

**DOI:** 10.1371/journal.pone.0205992

**Published:** 2018-11-20

**Authors:** Fujian Song, Max O. Bachmann, Paul Aveyard, Garry R. Barton, Tracey J. Brown, Vivienne Maskrey, Annie Blyth, Caitlin Notley, Richard Holland, Stephen Sutton, Thomas H. Brandon

**Affiliations:** 1 Norwich Medical School, Faculty of Medicine and Health Science, University of East Anglia, Research Park, Norwich, United Kingdom; 2 Nuffield Department of Primary Care Health Sciences, University of Oxford, Radcliffe Primary Care Building, Radcliffe Observatory Quarter, Woodstock Road, Oxford, United Kingdom; 3 Institute of Public Health, University of Cambridge, Cambridge, United Kingdom; 4 Department of Health Outcomes and Behavior, Moffitt Cancer Center, Tampa FL, United States of America; TNO, NETHERLANDS

## Abstract

**Background:**

Previous studies have shown that smoking and smoking cessation may be associated with health-related quality of life (HRQoL). In this study, we compared changes in HRQoL in people who maintained abstinence with people who had relapsed to smoking.

**Methods:**

This was a secondary analysis of data from a trial of a relapse prevention intervention in 1,407 short-term quitters. The European Quality of Life -5 Dimensions (EQ-5D) measured HRQoL at baseline, 3 and 12 months. Smoking outcome was continuous abstinence from 2 to 12 months, and 7-day smoking at 3 and 12 months. We used nonparametric test for differences in EQ-5D utility scores, and chi-square test for dichotomised response to each of the five EQ-5D dimensions. Multivariable regression analyses were conducted to evaluate associations between smoking relapse and HRQoL or anxiety/depression problems.

**Results:**

The mean EQ-5D tariff score was 0.8252 at baseline. People who maintained abstinence experienced a statistically non-significant increase in the EQ-5D score (mean change 0.0015, P = 0.88), while returning to smoking was associated with a statistically significant decrease in the EQ-5D score (mean change -0.0270, P = 0.004). After adjusting for multiple baseline characteristics, the utility change during baseline and 12 months was statistically significantly associated with continuous abstinence, with a difference of 0.0288 (95% CI: 0.0006 to 0.0571, P = 0.045) between relapsers and continuous quitters. The only difference in quality of life dimensions between those who relapsed and those who maintained abstinence was in the proportion of participants with anxiety/depression problems at 12 months (30% vs. 22%, P = 0.001). Smoking relapse was associated with a simultaneous increase in anxiety/depression problems.

**Conclusions:**

People who achieve short-term smoking abstinence but subsequently relapse to smoking have a reduced quality of life, which appears mostly due to worsening of symptoms of anxiety and depression. Further research is required to more fully understand the relationship between smoking and health-related quality of life, and to develop cessation interventions by taking into account the impact of anxiety or depression on smoking.

## Background

Cigarette smoking remains the leading preventable cause of premature deaths globally [1]. Results of previous studies have indicated that smoking is negatively associated with quality of life [2, 3], and smoking cessation is associated with reduced depression and anxiety, and improved quality of life [4, 5]. A study found that the level of happiness in ex-smokers who stopped smoking for at least a year was similar to never smokers, and higher than current smokers [6]. However, it is uncertain whether trying and failing to quit increases the level of psychological distress and depression [7–10]. Some studies have reported a significant association between smoking relapse and mental health problems [11, 12], while another study found no greater tendency to relapse for quitters with worsened mental health [13]. Studies showing that relapse is associated with simultaneous changes in quality of life or mood cannot identify the causal direction between them, and these effects may work in both directions.

In a randomised controlled trial, we evaluated the effectiveness of different self-help booklets for preventing smoking relapse in people who had stopped smoking for four weeks, and found no differences between trial arms in smoking relapse at 12 months [14, 15]. Here we compared changes in health-related quality of life (HRQoL) in people who maintained abstinence with people who had relapsed to smoking. We also investigated whether anxiety or depression problems were associated with subsequent smoking relapse, or smoking relapse was associated with subsequent anxiety or depression problems.

## Methods

This is a secondary analysis of data from a randomised trial (ISRCTN36980856) for smoking relapse prevention [14, 15]. The study was approved for research ethics (UK National Research Ethics Service REC reference: 11/EE/0091, dated 20/04/2011), and all participants were provided detailed information about the study and signed the consent form before participating. Participants were recruited between August 2011 and June 2013, and the follow-up of participants was completed by July 2014. Note that the recruitment start date shown in the ISRCTN registry (01/06/2011) was incorrect, and we confirm that the participant recruitment actually started since August 2011 after the trial’s registration on 25/07/2011. The authors also confirm that all ongoing and related trials for this intervention are registered. The study included 1,407 carbon monoxide (CO) verified quitters at 4 weeks after quit dates in stop smoking clinics. The study excluded quitters who were pregnant, unable to read booklets in English, or younger than 18 years. Participants received either a set of educational booklets or a single leaflet to prevent smoking relapse, and were followed up at 3 and 12 months after the quit date. There was no difference between trial arms in the occurrence of the primary endpoint in the trial, prolonged abstinence from 4 to 12 months (assessed by self-report of no more than five cigarettes in total, and confirmed by CO<10ppm at the 12 month follow-up). In the current study, we used usual continuous abstinence as the smoking outcome, defined similarly to the primary endpoint but from baseline (1 month after the quit date) to the 12 month follow-up. In addition, we also collected data on 7-day self-report smoking at 3 months and 12 months post quit date.

The European Quality of Life -5 Dimensions- 3 Level (EQ-5D) instrument was used to estimate levels of HRQoL [16]. Participants were asked to complete the EQ-5D questions at baseline (1 month after the quit date), and at 3 and 12 months after the quit date. EQ-5D instrument has 5 dimensions: mobility, self-care, usual activity, pain/discomfort, and anxiety/depression. For each dimension, there are three possible responses: “no problems”, “some or moderate problems”, and “severe or extreme problems”. In the current study, EQ-5D data were analysed using two methods; utility scores based on EQ-5D and dichotomisation of responses to each of the five EQ-5D dimensions.

Responses to EQ-5D dimensions were converted into a utility score (a scale where death is equal to 0 and full health 1) using the York A1 tariff [17,18]. The York A1 tariff enables a value to be assigned to each EQ-5D health state, where these scores are based on the preferences of a large sample of the UK population. Because the EQ-5D scores are not normally distributed, differences in EQ-5D scores between groups were tested using nonparametric Mann-Whitney test (two sample Wilcoxon rank-sum test). For ordered groups, we used the nonparametric test for trend (an extension of the Wilcoxon rank-sum test) [19]. We also calculated changes in utility score for individual participants between baseline and 3 or 12 months. Although the distribution of EQ-5D original scores was asymmetric, data on changes in utility score from baseline to the follow-up time point was symmetrically distributed. A previous study compared different methods and found that ordinary least squares (OLS) regression model was acceptable for the analysis of EQ-5D tariff data [2]. Therefore, as the main analysis, we performed OLS linear regression analyses to estimate the relationship between the change in utility score, as outcome, and smoking status as explanatory variable, after adjusting for multiple baseline variables. The baseline variables adjusted in the analyses were age, gender, married or living with a partner, unemployed or not, low level of education, free prescription, and the first cigarette within 5 minutes after waking before quitting.

The participants included in the current study were relatively healthy, with a low proportion of “severe or extreme problems” for individual EQ-5D dimensions. Therefore, responses to each of the EQ-5D dimensions were dichotomised into two categories: “no problems” and “some or severe problems” by combining “some to moderate problems” and “severe or extreme problems” [2]. We used chi-square test to analyse the association between the smoking outcome and problems in each of the five EQ-5D dimensions. Because differences in HRQoL scores were attributable mainly to problems in the anxiety/depression dimension, we explored the association between changes in smoking status and changes in anxiety/depression problems.

Stata/IC version 13.1 was used for statistical analyses, and statistical significance was defined as two sided P<0.05.

## Results

The main characteristics of study participants are shown in Table 1. The mean age of the participants was 47.8 years (SD 13.8). Of the 1,407 participants, 52.7% were females, 61.6% married or living with a partner, 52.6% in paid employment, 32.1% with A level or above education, and 56.3% in receipt of free prescription (free prescription is defined in Table 1). In terms of smoking history, 55.5% of the participants smoked 20 or more cigarettes daily and 42.2% smoked the first cigarette after waking up before the current quit attempt. Of the participants, 88.9% had previously attempted to quit and 71.6% had managed to stop smoking for more than 4 weeks before. The proportion of participants living with a smoking partner was 18.3%.

**Table 1:** The main characteristics of study participants.

Baseline variables	% (N = 1407)	Baseline EQ-5D utility score: mean (SD)	P value
Total	100%	0.8252 (0.2594)	
Age (years):			
Up to 39	29.5%	0.9042 (0.1823)	P_trend_<0.001
40–59	45.8%	0.8028 (0.2817)	
60 & above	24.7%	0.7701 (0.2739)	
Sex:			
Female	52.7%	0.8153 (0.2681)	P_m-w_ = 0.084
Male	47.3%	0.8362 (0.2490)	
Marital status:			
Married/living with partner	61.6%	0.8390 (0.2524)	Reference
Separated/divorced	15.9%	0.7742 (0.2922)	P_m-w_<0.001
Single	18.2%	0.8422 (0.2361)	P_m-w_ = 0.876
Other/unknown	4.3%	0.7426 (0.2906)	P_m-w_<0.001
Ethnic origin:			
White	98.4%	0.8248 (0.2602)	P_m-w_ = 0.472
All other	3.6%	0.8485 (0.2080)	
Employment status:			
In paid employment	52.6%	0.9025 (0.1692)	Reference
Unemployed	10.0%	0.7132 (0.3007)	P_m-w_<0.001
Looking after the home	7.4%	0.8229 (0.2685)	P_m-w_ = 0.002
Retired	20.3%	0.7515 (0.2861)	P_m-w_<0.001
Full time student	1.2%	0.8918 (0.1720)	P_m-w_ = 0.780
Other	8.5%	0.6480 (0.3890)	P_m-w_<0.001
Education:			
Degree or equivalent	15.2%	0.8610 (0.2172)	P_trend_<0.001
A level or equivalent	16.9%	0.8374 (0.2570)	
GCSE or equivalent	34.1%	0.8477 (0.2425)	(excluded
Other	12.4%	0.7924 (0.2926)	“unknown”)
None	20.0%	0.7627 (0.2877)	
Unknown	1.3%	0.8414 (0.2458)	
Free prescription*:			
Yes	56.3%	0.7702 (0.2947)	P_m-w_<0.001
No	43.7%	0.8989 (0.1791)	
Cigarettes ≥20/day before quitting			
Yes	55.5%	0.8086 (0.2750)	P_m-w_ = 0.020
No	44.5%	0.8457 (0.2371)	
First cigarette after waking up:			
In 5 minutes	42.2%	0.7848 (0.2924)	P_trend_<0.001
6–30 minutes	42.6%	0.8496 (0.2384)	
>30 minutes	15.2%	0.8678 (0.1987)	
Previous quit attempts:			
None	11.1%	0.8622 (0.2289)	P_trend_ = 0.04
1 attempt	26.2%	0.8234 (0.2528)	
2 attempts	23.7%	0.8327 (0.2718)	
3 or more	39.0%	0.8123 (0.2633)	
Longest quit time before:			
Not applicable	11.2%	0.8622 (0.2289)	P_trend_ = 0.008
<1 week	6.3%	0.8311 (0.2634)	
1–4 weeks	10.9%	0.8496 (0.2506)	
>4 weeks, <6 months	33.1%	0.8210 (0.2656)	
> = 6 months	38.5%	0.8115 (0.2631)	
Living with a smoking partner:			
Yes	18.3%	0.8327 (0.2530)	P_m-w_ = 0.656
No	81.7%	0.8235 (0.2609)	

P_trend_−the nonparametric test for trend across ordered groups (an extension of the Wilcoxon rank-sum test. P_m-w_−Mann-Whitney test (two sample Wilcoxon rank-sum test).

* Free prescription—The charge for a single prescription is £8.05 in the UK. Some people are entitled to free prescriptions because of their age (60 or over, or under 16, or aged 16 to 18 in full-education), income (on Income Support or qualified via other benefits or tax credits), or medical condition. GCSE–The General Certificate of Secondary Education.

The number of people who completed the EQ-5D-3L questionnaire was 1348 (95.8%) at baseline, 1290 (91.7%) at the 3 month follow-up and 1171 (83.2%) at the 12 month follow-up. The mean EQ-5D tariff score was 0.8252 at baseline. In bi-variable analyses, EQ-5D scores at baseline were statistically significantly associated with age, divorced or separated, employment status, education level, in receipt of free prescription, heavy smokers before quitting, and previous quit history (Table 1). Results of multivariable regression analysis found that EQ-5D scores at baseline were independently and negatively associated with age (P<0.001), being female (P = 0.002), unemployment (P<0.001), and first cigarette within 5 minutes of waking (P<0.001), after adjusting for other baseline characteristics.

After excluding three participants who died before the 12 month follow up, the proportion of continuous abstinence from baseline to 12 months was 32.4%. The mean EQ-5D utility scores at baseline, the 3 and 12 month follow up by smoking outcome are shown in Table 2. The differences in EQ-5D utility score between relapsers and continuous quitters were statistically non-significant at baseline and 3 months, but were statistically significant at 12 months (P = 0.040). The mean change in EQ-5D utility score between baseline and 12 months was -0.0270 (significantly deteriorated, P = 0.004) for relapsers and 0.0015 (non-significantly improved, P = 0.883) for continuous quitters. After adjusting for multiple baseline characteristics, the utility change during baseline and 12 months was statistically significantly associated with continuous abstinence, with a difference of 0.0288 (95% CI: 0.0006 to 0.0571, P = 0.045) between relapsers and continuous quitters.

**Table 2:** Mean EQ-5D utility scores at baseline and follow-ups by smoking status.

Time point	All participant		Abstinent at 12 months		Relapsed at 12 months		P value
	N	Mean	n	Mean	n	Mean	
Mean utility scores at different time points							
Baseline	1348	0.8252	442	0.8289	903	0.8234	0.412
At 3 months	1290	0.8255	451	0.8247	836	0.8261	0.873
At 12 months	1171	0.8072	451	0.8307	720	0.7924	0.040

Two sample Wilcoxon rank-sum (Mann-Whitney) test was used for the test of mean utility scores at different time points, and t-test for the change in utility scores.

**Fig 1:**
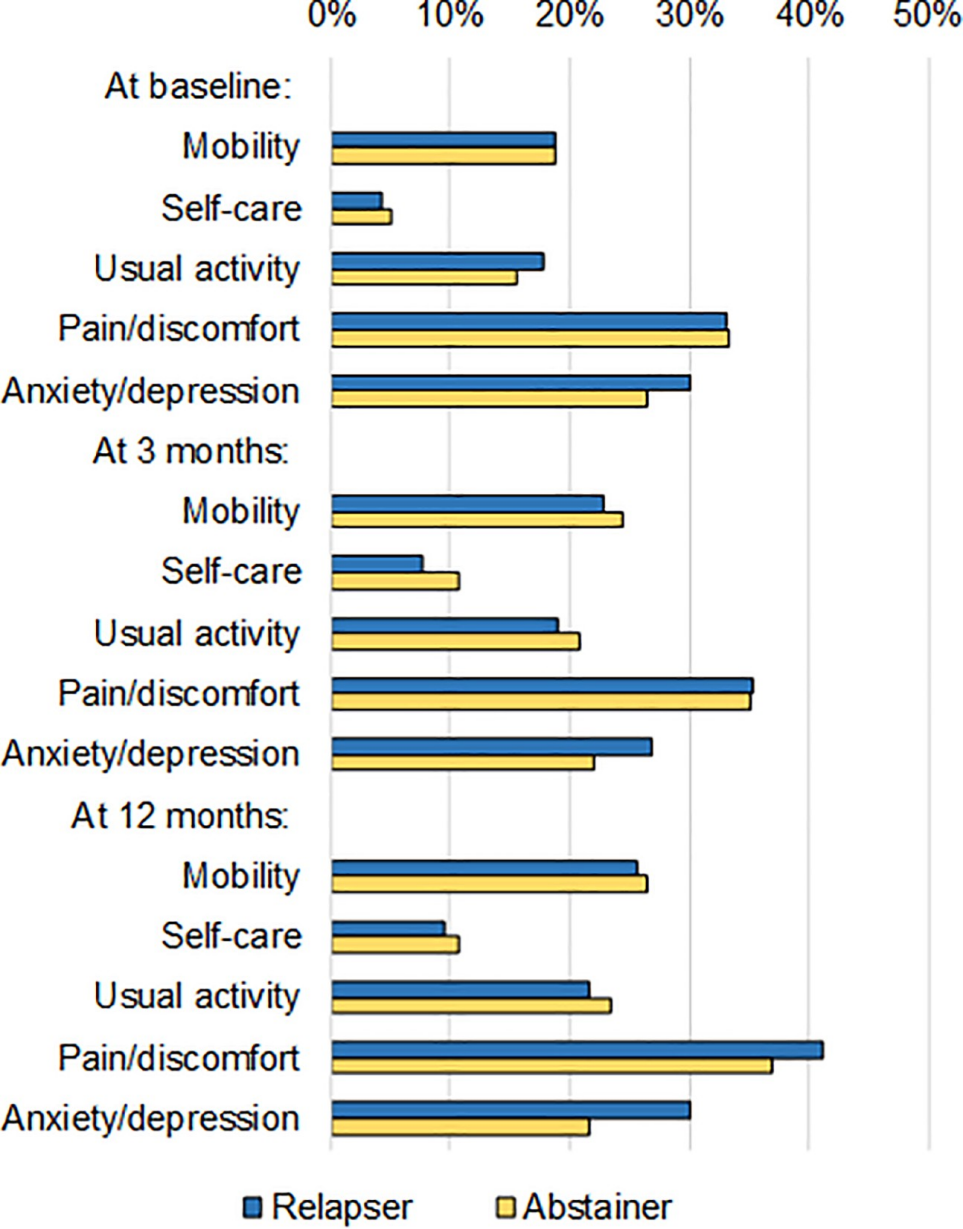
Proportion of participants with some/severe problems in different EQ-5D dimensions by continuous abstinence. In each EQ-5D dimension the “some problems” and “severe problems” were combined as “some/severe problems”. Differences in “some/severe problems” between the relapse and abstinent was statistically significant only for anxiety/depression dimension at 12 months (P = 0.001).

For specific EQ-5D dimensions, smoking status at 12 months was not statistically significantly associated with problems in mobility, self-care, usual activity, and pain or discomfort at baseline and the follow-ups (Fig 1). Of the five EQ-5D dimensions, only problems in anxiety/depression at 12 months was statistically significantly associated with smoking relapse (30% for relapses vs. 22% for continuous quitters, P = 0.001).

The relationships between smoking reported at the two follow-ups and some/severe problems in anxiety or depression dimension are shown in Table 3. Anxiety/depression problems at baseline were not significantly associated with smoking relapse at 3 months (P = 0.112) or at 12 months (P = 0.841), and anxiety/depression problems at 3 months were not significantly associated with smoking relapse at 12 months (P = 0.354). Smoking relapse at 3 months was not statistically significantly associated with anxiety/depression problems at 12 months (OR 1.22, 95% CI 0.87 to 1.71; P = 0.246). However, smoking relapse at 3 or 12 months was statistically associated with anxiety/depression problems at the same time points (Table 3).

**Table 3:** Results of logistic regression analyses of associations between anxiety or depression problems and smoking status at baseline, 3 and 12 months.

Any anxiety/depression problems	Odds ratio (95% confidence interval); P value	
	Smoking at 3 months	Smoking at 12 months
At baseline	1.27 (0.95–1.70); P = 0.112	1.03 (0.80–1.31); P = 0.841
At 3 months	2.28 (1.63–3.17); P<0.001	1.13 (0.87–1.47); P = 0.354
At 12 months	1.22 (0.87–1.71); P = 0.246	1.49 (1.14–1.95); P = 0.003

Logistic regression analysis using smoking at 3 or 12 months as the dependent variable, and adjusted for age, sex, married or not, unemployment, low education (GCSE or lower), receipt with free prescription, and the first cigarette in 5 minutes after waking up.

**Fig 2:**
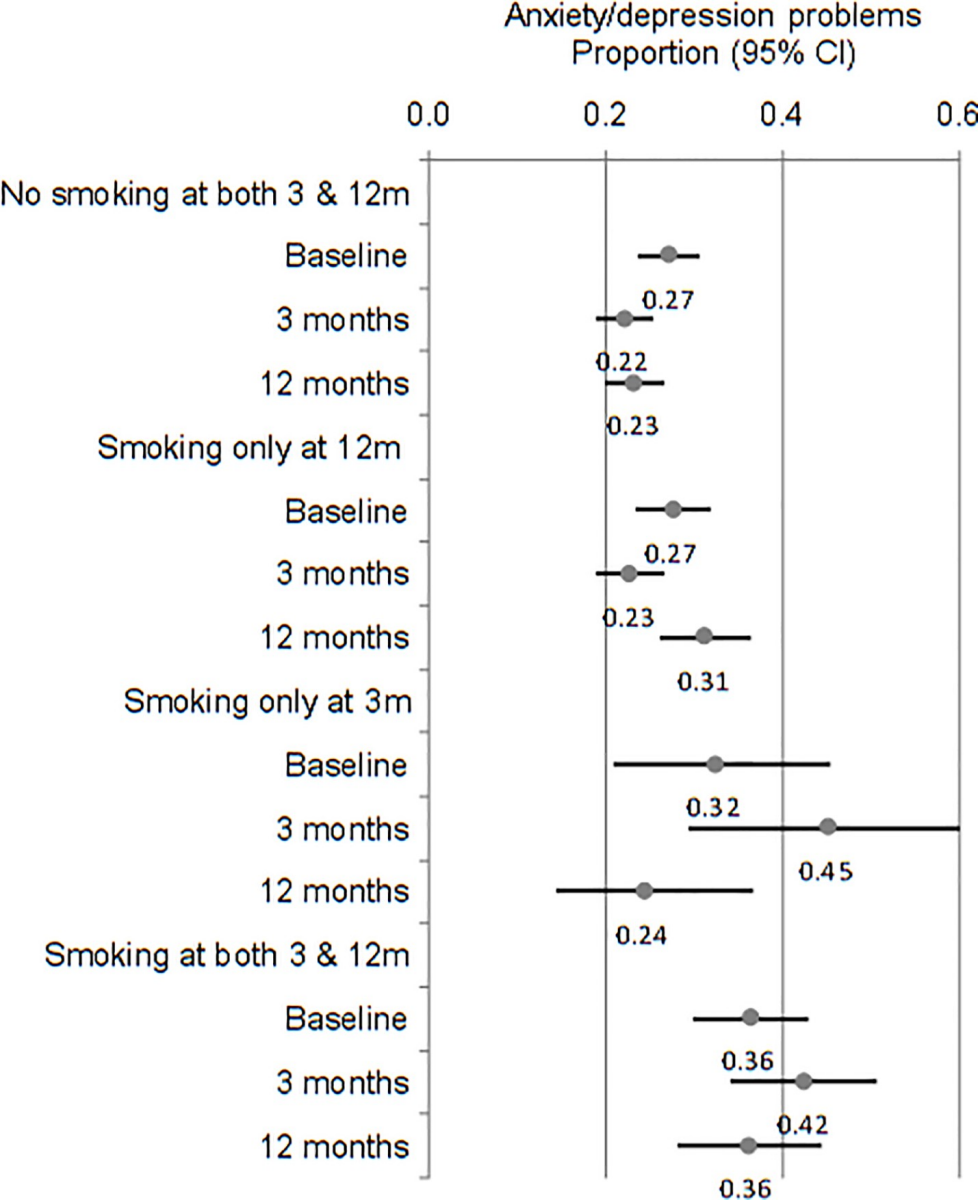
Proportions of people with anxiety/depression problems by any smoking during the 7-days before the 3-month and 12-month follow-up.

Fig 2 shows the proportion of participants with anxiety/depression problems by further subgrouping smoking status at baseline, 3 and 12 months. The proportion of anxiety/depression problems among participants who remained abstinent at both 3 and 12 months was 27% at baseline, and somewhat reduced to 22% at 3 months and 23% at 12 months. For participants who did not smoke at 3 months but smoked at 12 months, the proportion of anxiety/depression problems was higher at 12 months (31%), compared with that at baseline (27%) and 3 months (23%). It is interesting to note the fluctuation of anxiety/depression problems among participants who smoked at 3 months and did not smoke at 12 months; the proportion of anxiety/depression problems was somewhat high at baseline (32%), considerably increased at 3 months (45%), and then much reduced at 12 months (24%). Participants who reported smoking at both 3 and 12 months had a high proportion of anxiety/depression problems at baseline (36%) and at the two follow-ups (42% and 36% respectively).

## Discussion

In the current study, the mean EQ-5D utility score was 0.8252 (SD 0.2594) at baseline and 0.8072 (SD 0.2456) at 12 months, which is similar to the score reported for a representative sample of English former smokers, 0.8225 (SD 0.2512) [2]. It was found that EQ-5D utility at baseline was negatively associated with certain participant characteristics, including older age, being female, unemployed, and heavy smoking before quitting. At follow-up, those who had relapsed had a lower quality of life, while those maintaining abstinence had a small and non-significant increase in HRQoL. The difference in HRQoL between the relapsers and continuous quitters at follow-up was statistically significant, and due to changes in anxiety or depression, with no evidence of changes in other EQ-5D dimensions.

A study of the English general population found that heavy smokers had poorer HRQoL in all five EQ-5D dimensions compared with never smokers [2]. In the current study of short-term quitters, analyses of EQ-5D dimensions revealed that the differences in HRQoL between relapsers and continuous quitters were mainly attributable to problems in the anxiety/depression dimension. This finding was consistent with those from a previous study that examined the relationship between smoking cessation and HRQoL using the EQ-5D [20]. The association between anxiety/depression and smoking or smoking cessation is complex, and results from different studies are often inconsistent. For example, a recent study did not find a long-term impact of smoking cessation on depression, although depression reduced possibility of quitting [21]. Because smokers often use cigarettes to attempt to cope with stress and anxiety, many smokers and some health professionals mistakenly believe that smoking cessation will worsen stress and anxiety symptoms [22]. This belief may arise because tobacco withdrawal symptoms include anxiety and depression immediately after abstinence, although withdrawal symptoms last only a few weeks [23]. However, many previous studies found that failed quit attempts may increase anxiety or depression [7–9, 24, 25], and successful smoking cessation reduces stress and anxiety [4–6, 22].

A prospective study published in 1990 examined the relation between changes in stress levels and changes in smoking status, and concluded that the relation was possibly bidirectional [5]. Although the association between changes in smoking status and changes in stress levels was consistently observed in other studies [22, 26], the causal direction remains uncertain. In a recent study by Taylor and colleagues [13], deterioration in mental health after achieving and maintaining abstinence until four months did not predict relapse to smoking by 12 months. In the current study, we found that smoking relapse was accompanied with increased anxiety or depression problems measured at the same time. However, anxiety or depression problems at baseline or at 3 months were not associated with smoking relapse at 12 months (Table 3, Fig 2). It is also interesting to note that relapse to smoking by 3 months was associated with an increase in anxiety and depression at that time, while returning to abstinence by 12 months was associated with reduced anxiety and depression at 12 months (Fig 2).

The effects of anxiety or depression on smoking relapse may occur promptly with little delay, so that smoking is closely associated with the present, rather than the previous, mental status. This may explain why deterioration in mental health did not predict smoking relapse after eight months in a previous study [13]. However, as a failed quit attempt, relapse to smoking itself is likely a stressful incident. In contrast to the immediate effects of anxiety and depression on smoking relapse, the beneficial effects of smoking cessation on anxiety or depression may not be perceived immediately and may often be complicated by withdrawal symptoms. Consequently, people with mental illness have a higher smoking prevalence and are less likely to quit than people without mental illness [27]. Although accumulating evidence suggests that smoking cessation improves mental health and reduces stress or depression in long term, more efforts are required to manage stress and other depressive symptoms in smoking cessation interventions, whilst emphasising these potential longer mental health benefits.

### Strengths and limitations

This is the first study to examine changes in EQ-5D based HRQoL in a large number of people who had quit smoking for several weeks after receiving smoking cessation treatment. The study provided data on EQ-5D based utility scores by smoking relapse status, which will help evaluations of long-term cost-effectiveness and cost-utility of smoking cessation and tobacco control interventions [28]. By measuring EQ-5D status at baseline, 3 and 12 months, and collecting data on smoking status at 3 and 12 months, we were able to investigate the association between changes in smoking status and changes in HRQoL (specifically, changes in anxiety/depression problems).

We found that the difference in changes in HRQoL between relapsers and continuous quitters was statistically significant, although the clinical and public health importance of the difference was unclear. We focused on the association between smoking relapse and HRQoL in the present study. However, it should be stressed that smoking relapse is also associated with other demographic or socioeconomic factors, including age, marital status, employment status, and living with a partner who smokes [14]. The participants of the current study were predominantly white (98%), reflecting the ethnic composition of the area; pregnant women, people younger than 18 years and those who were unable to read English were excluded [14], so that the results of the current study may not be generalizable to these groups. As mood and smoking status were assessed infrequently, this study alone cannot clarify the causal pathway. The current study was observational in nature, and we could not rule out the existence of other factors or events that were associated with both relapse to smoking and changes in EQ-5D responses.

### Conclusions

Relapse to smoking is associated with reduced quality of life measured by EQ-5D, which seems mainly attributable to problems of anxiety and/or depression. Further research is required to more fully understand the relationship between smoking and health-related quality of life, and to develop cessation interventions by taking into account the impact of anxiety and depression on smoking.

## Supporting information

S1 Dataset Data from the SHARPISH trial for smoking relapse and health-related quality of life. (TXT)
